# Effect of adhesions on laparoscopically-assisted vaginal hysterectomy outcome: a 10-year retrospective, comparative study of 1683 consecutive cases

**DOI:** 10.1007/s00404-026-08396-1

**Published:** 2026-03-19

**Authors:** Luz Angela Torres-de la Roche, Alina Jara Schulte, Rudy Leon De Wilde, Rajesh Devassy, Garri Tchartchian, Harald Krentel, Maya Sophie de Wilde

**Affiliations:** 1 University Hospital for Gynecology, Pius Hospital, University Medicine Oldenburg, Carl Von Ossietzky University, 26121 Oldenburg, Germany; 2 Special Interest Group Adhesions Research of the European Society for Gynaecological Endoscopy, Dubai, UAE; 3 Clinic Für Minimal Invasive Surgery, 14129 Berlin, Germany; 4 Department of Obstetrics and Gynecology, Klinikum Aschaffenburg-Alzenau, Aschaffenburg, Germany

**Keywords:** Laparoscopically-assisted vaginal hysterectomy, LAVH, Benign gynaecological conditions, Intraoperative complications, Adhesions

## Abstract

**Introduction:**

Hysterectomy is a frequently employed treatment modality in gynaecological diseases. In the context of various approaches to vaginal hysterectomy, laparoscopically-assisted vaginal hysterectomy (LAVH) could eventually improve the safety in cases where patients without genital prolapse present with preoperative peritoneal adhesions. The present analysis examined intraoperative and immediate postoperative adhesions-related outcomes.

**Methods:**

Monocentric, comparative, retrospective study of a single cohort of women without genital prolapse who underwent LAVH for benign gynaecological conditions between January 2010 and December 2019. Patients without peritoneal adhesions were compared with patients with adhesions, as diagnosed at the beginning of the procedure. Mann–Whitney-U test was used for the comparative analysis.

**Results:**

Among 1,638 patients, 562 patients (34.3%) had preoperative adhesions. Main indications were for myoma (71%) and adenomyosis (14.9%). The mean operation time in the adhesion group was significantly longer than in the no-adhesion group (106 ± 44 min vs. 90 ± 35 min; p < 0.001). Adhesiolysis was required in 88% within the adhesion group (n = 495). No significant differences were observed regarding mean estimated intraoperative blood loss (87 ± 100 ml vs. 90 ± 95 ml; p = 0, 418), uterine weight (220 ± 227 g vs. 230 ± 203 g; p = 0, 38), or morcellation (52% vs. 55.8%; p = 0, 142). Most patients in both groups did not experience complications (95% vs. 97.2%).

Laparoconversion due to technical difficulties in performing the endocopic surgery or due to the presence of adhesions was rarely needed (1.6% vs. 0.6%). The intraoperative complication rate was low but significantly different in both groups (2% vs. 0.7%; p = 0.02), including bowel injuries (n = 6 vs. n = 3) and urinary bladder injuries (n = 4 vs. n = 5). %), which were diagnosed immediately and treated successfully. The postoperative complication rate was similar in both groups (3.4% vs. 2.1%; p = 0.138), mainly urinary tract infection. All adverse events were treated successfully; second laparoscopic surgery was conducted in five patients with adhesions and in nine patients without adhesions for haematoma removal.

**Conclusion:**

LAVH could be considered a safe and feasible surgical approach for women without genital prolapse who have preoperative peritoneal adhesions and require vaginal hysterectomy for benign gynaecological conditions. This approach facilitates the localisation and treatment of adhesions at the commencement of surgery, as well as the control of possible injuries that may arise during the procedure, and is associated with a low complication rate.

## What does this study adds to the clinical work

**Table:** 

Women who do not present with uterine prolapse at the time of total hysterectomy for benign uterine diseases may benefit from laparoscopically assisted vaginal hysterectomy (LAVH) due to its lower complication rates and shorter recovery periods.	

## Introduction

Hysterectomy is one of the most frequently performed operations worldwide [1, 2], recommended in benign gynaecological diseases. Various approaches were developed over time. Minimally-invasive techniques are preferable to abdominal hysterectomy (AH) since laparotomy is more invasive, associated with higher complication rates including ureteric lesions as well as longer operation time and prolonged recovery [3–6]. However, AH is still the most applied technique worldwide [7].

Among minimally-invasive approaches, vaginal hysterectomy (VH) is reported to be superior regarding operation time, blood loss, hospitalization, recovery time, and overall costs [1, 3, 4, 7–13]. In comparison, laparoscopically-assisted vaginal hysterectomy (LAVH) enables a more profound exploration of the pelvis as well as additional laparoscopic interventions (such as adhesiolysis, removal of endometriotic lesions, adnexectomy etc.). It is associated with less complications and less postoperative pain compared to AH [9, 11, 12, 14–17]. The risk of intraoperative haemorrhage during LAVH decreases with rising level of surgical experience and can increase with increasing uterus size [18–20]. However, few studies on the feasibility of LAVH for benign gynaecological conditions in women without genital prolapse who have preoperative peritoneal adhesions and require hysterectomy for benign gynaecological conditions have been published. With surgical procedures developing constantly, an analysis of the outcomes of this surgical approach could provide new and interesting results and contributes to further improving the treatment of this group of patients.

## Methods

In this retrospective monocentric study, data were collected of all patients who consecutively underwent LAVH as treatment for benign gynaecological conditions without genital prolapse at the University Hospital for Gynaecology, Pius-Hospital Oldenburg, Germany, in a period of 10 years, between January 2010 and December 2019. Prior to operation, written informed consent was obtained from all patients.

Inclusion criteria included: ≥ 18 years old, LAVH as recommended surgical approach, benign gynaecological condition as indication for LAVH (including uterine myoma, other benign neoplasia of the female genital organs, endometriosis, chronic pelvic pain, abnormal uterine bleeding, menopausal symptoms, uterine or cervical dysplasia), no genital prolapse, surgery performed between 01/01/2010 and 31/12/2019. Exclusion criteria included: suspected or confirmed cancer diagnosis, acute infection of the pelvic organs prior to surgery, genital prolapse, postoperative complications or post-partum complications as primary indication for LAVH, coagulation disorder and/or anticoagulation therapy during the last six months prior to surgery.

This retrospective data analysis was approved by the Institutional Review Board of Pius-Hospital Oldenburg and by the Ethics Committee of the Carl von Ossietzky University Oldenburg, Germany (2020–084) before initiation.

### Data collection and statistical analysis

Cases were identified by the relevant ICD-10-codes (International Classification of Diseases) for benign gynaecological conditions (excluding genital prolapse) as well as the relevant OPS-codes (Operation and Procedure Classification System) for LAVH. All variables of interest were collected from the Orbis database and the digital archive of the Pius-Hospital. Anonymized case data were transferred to SPSS (software version 29.0) for statistical analysis.

Descriptive statistics were used for quantitative or numerical variables, including central tendency measures (percentage, mean, median or mode) and dispersion measures (standard deviation or ranges). The comparative analysis was conducted using Welch’s t-test for comparing means, Pearson’s Chi-squared test for categorical variables, and logistic regression analysis and Mann–Whitney U test for multivariable analysis. A p-value less than 0.05 was designated as significant.

### LAVH standard technique

- After positioning of the patient, desinfection and sterile draping of the operation situs, the uterus is examined by means of palpation and eventually hysteroscopy. In case the hysteroscopy shows no signs of malignancy, LAVH is conducted as follows.
- The operation starts by insufflating carbon dioxide into the abdomen and inserting the optical system as well as all necessary trocars. Afterwards, the abdominal cavity gets thoroughly examined. Special attention is paid to adhesions and endometriotic lesions. The outcome of the examination determines on further procedures (e.g. adhesiolysis, removal of endometriotic nodules, and guideline-based surgery of adnexal tumours).
- Eventually, severing the ligamentum rotundum to increase the mobility of the uterus.
- Preparation is conducted by means of bipolar coagulation and endoscopic scissors. In the course of LAVH, it ends above the uterine artery which is severed thereafter through vaginal approach after freeing the ovaries and tubes laparoscopically.
- Opening the peritoneum of the plica vesicouterina and shifting the urinary bladder can facilitate the vaginal part of LAVH in case of previous C-sections or local uterine myoma.
- The cervix is incised vaginally circularly, and the urinary bladder is shifted in the cranial direction. The vesicouterine and rectouterine excavations are opened by scissors, and the uterus is separated from the sacrouterine ligaments and the parametrium by ligating the vessels and surrounding tissue.
- Thereafter, the uterus is removed vaginally. In case of bulky specimens, the uterus can be morcellated. During morcellation, the uterus is cut into smaller pieces with a scalpel before removal
- In case of visible pathological anomalies, the ovaries and tubes can be resected; otherwise, they are left.
- The vagina is closed with interrupted sutures. A vaginal tamponade is applied to prevent bleeding and removed 24 h later. The urinary tract is checked for integrity by catheterizing and cystoscopy.
- A second intraoperative laparoscopy is performed for the purpose of controlling potential injuries to nearby organs and intraabdominal bleeding.
- After desufflating the pneumoperitoneum, trocar incisions are sealed with single sutures and wound dressings are applied.

## Results

1638 patients were eligible for data collection. 562 patients (34.3%) had preoperative adhesions; 1076 patients (65.7%) showed no adhesions at time of surgery (Table 1).

**Table 1:** Patient characteristics in LAVH patients with and without preoperative peritoneal adhesions

Variable	Results of patients with adhesions (n = 562)		Results of patients without adhesions (n = 1076)	
Age (years)	Mean: 47 (SD ± 7)	Range: 28–79	Mean: 46 (SD ± 6)	Range: 25–76
BMI (kg/m^2^)	Total (n = 562)	%	Total (n = 1076)	%
< 18.5	5	0.9	16	1.5
18.5–24.9	201	35.8	430	40.0
25.0–29.9	168	29.9	316	29.4
30.0–34.9	107	19.0	170	15.8
35.0–39.9	47	8.4	80	7.4
> 40.0	32	5.7	57	5.3
No data	2	0.4	7	0.7
Menopausal status	Total (n = 562)	%	Total (n = 1076)	%
Reproductive	428	76.2	853	79.3
Perimenopausal	55	9.8	107	9.9
Postmenopausal	48	8.5	67	6.2
No data	31	5.5	49	4.6
Parity	Total (n = 562)	%	Total (n = 1076)	%
Nulliparous	123	21.9	215	20.0
1–2 children	319	56.8	637	59.2
3–4 children	117	20.8	215	20.0
6–8 children	3	0.5	6	0.6
> 8 children	0	0	3	0.3
Prior abdominal/pelvic surgery	Total (n = 562)	%	Total (n = 1076)	%
None	90	16.0	412	38.3
1–2	300	53.4	481	44.7
3–5	147	26.2	147	13.7
6–8	18	3.2	15	1.4
> 8	4	0.7	10	0.9
No data	3	0.5	11	1.0

Patients with adhesions (adhesion group) ranged in age from 28 to 79 years (mean age: 47 ± 7 years); 35.8% (n = 201) had a normal weight and 33.1% (n = 186) were obese (BMI > 30 kg/m^2^). At time of diagnosis, most patients were still within their reproductive-age years (n = 428; 76.2%), had given birth prior to surgery (n = 439; 78.1%) and had undergone an abdominal or pelvic surgery previously (n = 469, 83.5%).

Patients without adhesions (no-adhesion group) were between 25 and 76 years old (mean age: 46 ± 6 years). The majority (n = 446; 41.4%) was of normal weight and almost a third (n = 307; 28.5%) was obese (BMI > 30 kg/m^2^). At time of diagnosis, most patients were in their reproductive-age years (n = 853; 79.3%), had given birth prior to surgery (n = 861; 80.0%) and had received abdominal and/or pelvic surgery previously (n = 653; 60.0%).

Regarding surgical outcomes (Table 2), more than 90% of patients with adhesions (n = 513) underwent LAVH without adnexectomy; most of them had myomatosis disease (n = 399; 71.0%) or adenomyosis (n = 84; 14.9%). Adhesiolysis was performed in almost 90% (n = 495). The mean incision-suture time was 106 min (SD ± 44) with the longest operation lasting 8.5 h (515 min). The mean estimated intraoperative blood loss was 87 ml (SD ± 100), the largest amount being 1.5 L. The mean uterine weight was 220 g (SD ± 227), the largest uterus weighted at 3,073 g. In more than 50% (n = 292), the uterus was morcellated during the operation. Laparoconversion was conducted in nine cases (1.6%), mostly because of large uterine size or severe adhesions.

**Table 2:** Surgical outcomes of LAVH patients with and without preoperative peritoneal adhesions

Variable	Results of patients with adhesions (n = 562)		Results of patients without adhesions (n = 1076)	
Surgery method: LAVH	Total (n = 562)	%	Total (n = 1076)	%
without adnexectomy	513	91.3	1030	95.7
with unilateral adnexectomy	15	2.7	10	0.9
with bilateral adnexectomy	34	6.0	36	3.3
with adhesiolysis	495	88.1	/	/
Incision-suture time (min)	Total (n = 562)	Range/%	Total (n = 1076)	%
Mean	106 (SD ± 44)	Range: 37–515	90 (SD ± 35)	Range: 25–327
≤ 90	236	42.0	621	57.7
91–120	182	32.4	297	27.6
121–150	82	14.6	95	8.8
151–180	29	5.2	41	3.8
> 180	33	5.9	21	2.0
No data	0	0	1	0.1
Estimated intraoperative blood loss (ml)	Total (n = 562)	Range/%	Total (n = 1076)	%
Mean	87 (SD ± 100)	Range: 0–1500	91 (SD ± 84)	0–1200
≤ 100	449	79.9	858	79.7
101–200	88	15.7	163	15.1
201–300	13	2.3	24	2.2
301–500	2	0.4	10	0.9
> 500	4	0.7	5	0.5
No data	6	1.1	16	1.5
Uterine weight (g)	Total (n = 562)	Range/%	Total (n = 1076)	%
Mean	220 (SD ± 227)	Range: 25–3073	232 (SD ± 195)	14–1892
10–160	304	54.1	493	45.8
161–320	161	28.6	367	34.1
321–480	51	9.1	107	9.9
481–640	24	4.3	58	5.4
> 640	20	3.6	48	4.5
No data	2	0.4	3	0.3
Morcellation	Total (n = 562)	%	Total (n = 1076)	%
Not conducted	269	47.9	474	44.1
Conducted	292	52.0	600	55.8
No data	1	0.2	2	0.2
Intraoperative conversion of surgery method	Total (n = 562)	%	Total (n = 1076)	%
No conversion	553	98.4	1,066	99.1
Laparoconversion	9	1.6	6	0.6
Conversion to VH	0	0	2	0.2
Conversion to TLH	0	0	2	0.2
Pathological diagnosis	Total (n = 562)	%	Total (n = 1076)	%
No pathology documented	31	5.5	54	5.0
Myoma	399	71.0	786	73.0
Adenomyosis	84	14.9	133	12.4
Endometrial pathology	36	6.4	79	7.3
Cervical pathology	3	0.5	15	1.4
Ovarian pathology	9	1.6	9	0.8

Approximately 95% of patients without adhesions (n = 1030) underwent LAVH without adnexectomy; most of them had myoma (n = 786; 73.0%), followed by adenomyosis (n = 133; 12.4%). The mean incision-suture time was 90 min (SD ± 35) with the longest operation lasting 327 min. The mean estimated intraoperative blood loss was 91 ml (SD ± 84), the largest amount being 1.2 L. The mean uterine weight was 232 g (SD ± 195), the largest uterus weighted at 1,892 g. In more than 50% (n = 600; 55.8%), the uterus was morcellated during the operation. Laparoconversion was conducted in six cases (0.6%), mostly because of large uterine size or severe adhesions.

The results of Mann–Whitney-U test show a significant difference in the central tendencies of both groups (p < 0.001), indicating that a longer surgical time is to be expected in patients with peritoneal adhesions that undergo LAVH (effect size = medium; r = 0.2).

As shown in Table 3, data on intraoperative and postoperative complications revealed that 95% of cases in the adhesion group (n = 534) experienced no complications, resulting in an overall complication rate of 5%. Specifically, the rate was 1.6% (n = 9) for intraoperative complications, 3% (n = 17) for postoperative complications, and 0,4% (n = 2) for both intra- und postoperative complications. Intraoperative complications were bowel injuries (n = 6; 1.1%) and injuries of the urinary bladder (n = 5; 0.9%). No ureteric lesions occurred. In all 11 cases, the bowel or bladder injury was detected intraoperatively and successfully treated. Only two of these patients developed urinary tract infection postoperatively. Postoperatively, the most frequent complication in the adhesion group was urinary tract infection (n = 12; 2.1%) followed by haemorrhage (n = 6; 1.1%) and pneumonia (n = 1; 0.2%). In accordance with Clavien-Dindo Classification of Surgical Compliations [21], two cases of minor haemorrhage that did not receive further treatment were ranked grade I. 11 cases that required antibiotics were ranked grade II, and five cases that required second laparoscopic surgery under general anaesthesia for haematoma removal were ranked grade IIIb. One patient was transferred to the intensive care unit for machine-assisted ventilation due to severe pneumonia and was ranked grade IVa. No complication grade V was observed within the adhesion group.

**Table 3:** Intra- and postoperative complications of LAVH patients with and without preoperative peritoneal adhesions

Complication	Results of patients with adhesions (n = 562)		Results of patients without adhesions (n = 1076)	
Cases	Total (n = 562)	%	Total (n = 1076)	%
without complication	534	95.0	1,046	97.2
with intraoperative complications	9	1.6	7	0.7
with postoperative complications	17	3.0	23	2.1
with intra- and postoperative complications	2	0.4	0	0
Intraoperative complication	Total (n = 11; 2.0%)	%	Total (n = 7; 0.7%)	%
Bowel injury	6	54.5	3	42.9
Urinary bladder injury	5	45.5	4	57.1
Ureteric lesions	0	0	0	0
Injury sutured intraoperatively	11	100.0	7	100.0
Postoperative complication	2	18.2	0	0
Postoperative complication	Total (n = 19; 3.4%)	%	Total (n = 23; 2.1%)	%
Urinary tract infection	12	63.2	11	47.8
Haemorrhage	6	31.6	11	47.8
Pneumonia	1	5.3	0	0
Multiple complications	0	0	1	4.4
Clavien-Dindo classification				
Grade I (no treatment required)	2	10.5	3	13.1
Grade II (antibiotics)	11	57.9	11	47.8
Grade IIIb (haematoma removal under general anaesthesia)	5	26.3	9	39.1
Grade IVa (transfer to ICU due to pneumonia)	1	5.3	0	0

It was observed that most patients in the no-adhesion group did not experience complications (n = 1,046; 97.2%), resulting in an overall complication rate of 2.8% (n = 30). This included seven patients (0.7%) who were affected by intraoperative complications and 23 patients (2.1%) who developed postoperative complications. Intraoperatively, three patients without adhesions (0.3% of the group) experienced a bowel injury, and four patients (0.4% of the group) an injury of the urinary bladder, all of which were successfully identified intraoperatively and immediately treated. No ureteric lesions occurred and none of these patients developed postoperative complications. Postoperatively, 11 cases had urinary tract infection and haemorrhage. According to the Clavien-Dindo Classification of Surgical Complications [21], 11 patients who required antibiotics were ranked grade IIm and nine patients who required second laparoscopic surgery under general anaesthesia for haematoma removal were ranked grade IIIb. No complication grad IV or V were reported among the no-adhesion group.

The findings of the logistic regression analysis (Table 4) show a statistically significant difference in the incidence of intraoperative complications among patients with and without preoperative adhesions (p = 0.02). No statistically significant difference was observed between the postoperative complication rates (p = 0.138). However, the quality analysis performed to determine the importance of adhesions as a risk factor for both types of complications showed that this variable had little influence on these surgical outcomes (Cox & Snell R square = 0.003 for intraoperative and 0.001 for postoperative complications; Nagelkerkes R-square = 0.029 for intraoperative and 0.006 for postoperative complications).

**Table 4:** Logistic regression – Complication rates in LAVH patients with and without preoperative adhesions

Logistic regression	Results (n = 562 patients with adhesions, n = 1.076 without adhesions)		
	Overall complications (n = 28; n = 30)	Intraoperative complications (n = 11; n = 7)	Postoperative complications (n = 19; n = 23)
Omnibus test			
Chi-square	4.967	5.424	2.196
df	1	1	1
Sig	0.026	0.020	0.138
Model summary			
- 2 log-likelihood	496.486	192.768	388.457
Cox & Snell R-square	0.003	0.003	0.001
Nagelkerkes R-square	0.011	0.029	0.006
Variables in the equation			
Regression coefficient B	0.603	1.117	0.471
Standard error	0.268	0.486	0.314
df	1	1	1
Sig	0.24	0.022	0.134
Exp (B)	1.828	3.049	1.602

## Discussion

International studies advocate a reduction in hysterectomy by laparotomy procedures and recommend the active application minimally invasive techniques. They also recommend the use of LAVH cases were classical vaginal approach is not feasible due to the absence of organ prolapse [6, 9, 11, 15]. However, few studies on the feasibility of LAVH for women without genital prolapse who have preoperative peritoneal adhesions and require vaginal hysterectomy for benign gynaecological conditions have been published.

Preoperative peritoneal adhesions are known to complicate surgical interventions which can result in longer operation time, higher blood loss and higher complication rates [22, 23]. In this study, we retrospectively analysed data of 1638 consecutive LAVH cases in a period of 10 years and compare the outcomes of patients with and without preoperative adhesions.

Most patients in both groups were diagnosed with myomata, same as in other reports [1, 18]. In patients with adhesions, LAVH lasted averagely 106 min which was similar 16 min longer than in patients without adhesions (p =  < 0.001) but shorter compared to the data reported by Müller et al. (122 min) [24] and Litwińska et al. (131 min) [9]. In the present analysis, 180 min operation time was exceeded in 33 cases with adhesions (5.9%) and in 21 cases without adhesions (2%). The prolongation of surgery could have been caused by laparoconversion or by the occurrence of intraoperative complications, which were more frequent in patients with adhesions. Mann–Whitney-U-test showed that a longer operation time is, therefore, to be expected in patients with peritoneal adhesions that undergo LAVH.

The mean estimated intraoperative blood loss in patients with adhesions was 87 ml, thus similar to patients without adhesions (92 ml; p = 0, 418) and lower compared to data reported by Hwang et al. (343 ml) [16]. Only 0.7% of patients with adhesions lost more than 500 ml, only slightly more than patients without adhesions (0.5%). The heavy blood loss could be explained by the need for laparoconversion or by injuries to neighbouring organs during the procedure.

Complications occurred more often in patients with adhesions compared to patients without adhesions (5% compared to 2.8%; p = 0.03) and compared to a retrospective study including 184 cases by Dolanbay et al. from 2016 (2.7%) [18]. Intraoperative complications occurred more often in the adhesion group (2% vs. 0.7%; p = 0.02) and thus slightly more frequent than in the health report by AQUA (1.4%) [25] and the study by Shen et al. (1.3%) [19] but similar to the study by Song et al. (2.2%) [26]. Laparoconversion was conducted more frequently in patients with adhesions than in patients without adhesions (1.6% versus 0.6%) but less often compared to other studies (7.5% [4], 4.8% [26]). Bladder and bowel injuries were the sole intraoperative complications in the present cohort, and thus the same most frequent intraoperative adverse events as in other studies [1, 9, 19, 27]. The LAVH approach is known for its advantage to prevent injuries to nearby organs during hysterectomy, specifically ureteric lesions [5] which explains why these complications did not occur in any of the groups analysed in this study.

In contrast to the intraoperative complications, no statistically significant difference was observed in the rates of postoperative complications grade I to IV according to Clavien-Dindo Classification (3.4% versus 2.1%; p = 0.138), which were lower than those reported in the AQUA study [25] and in study by Müller et al. [24] (4% and 4.6% respectively). Urinary tract infection was the most frequent postoperative complication in all cases; no ureteric lesions occurred. Additional treatment was successful in all cases of adverse event. Most patients with adhesions who had postoperative complications did not require second surgery (n = 13 of 19; 68.4%). In all five cases of second laparoscopic surgery, only haematoma removal was required. The single patient with life-threatening pneumonia was successfully treated at the intensive care unit.

### Strengths and limitations of the study

The retrospective nature of this analysis resulted in limitations, primarily concerning the details of the reasons that led to the reported complications and the time required to perform laparoconversion. Because of the considerable number of cases that have been analysed, there results are of significance in terms of the international interest in the clinical outcomes that recommend LAVH as a preferred hysterectomy approach, also in cases with preoperatively existing adhesions.

## Conclusion

This analysis has been conducted to evaluate the quality of care provided to women without genital prolapse who underwent LAVH for benign gynaecological conditions who present preoperative peritoneal adhesions. Important surgical outcomes including appropriate procedure times, low blood loss, low complication rates and absence of ureteric lesions confirm that LAVH is a suitable and safe treatment option for patients with benign gynaecological conditions. Nevertheless, patients should be informed that preoperative adhesions can prolong surgical time and enhance iatrogenic complications without affecting the advantages of LAVH, which include minimal invasiveness, low postoperative pain and rapid convalescence. In addition, the pelvic exploration allows the possibility of additional interventions such as adnexectomy, adhesiolysis or removal of endometriotic lesions.

## Data Availability

The clinical data analysed in this study are stored at University Hospital for Gynaecology, Pius Hospital, University Medicine Oldenburg, Oldenburg 26,121, Germany. For data protection reasons, the repository of data related to this analysis is not publicly accessible.
